# IL28B SNP rs12979860 Is a Critical Predictor for On-Treatment and Sustained Virologic Response in Patients with Hepatitis C Virus Genotype-1 Infection

**DOI:** 10.1371/journal.pone.0018322

**Published:** 2011-03-30

**Authors:** Chun-Yen Lin, Ji-Yih Chen, Tsung-Nan Lin, Wen-Juei Jeng, Chien-Hao Huang, Chang-Wen Huang, Su-Wei Chang, I-Shyan Sheen

**Affiliations:** 1 Department of Gastroenterology and Hepatology, Linkou Medical Center, Chang Gung Memorial Hospital, Kweishan, Taoyuan, Taiwan; 2 Department of Rheumatology, Allergy and Immunology, Linkou Medical Center, Chang Gung Memorial Hospital, Kweishan, Taoyuan, Taiwan; 3 College of Medicine, Chang Gung University, Kweishan, Taoyuan, Taiwan; 4 Division of Biostatistics, Institute of Biomedical Sciences, Academia Sinica, Nankang, Taipei, Taiwan; Ulm University, Germany

## Abstract

**Background:**

Single nucleotide polymorphisms (SNPs) of *interleukin-28B (IL28B)* have received considerable interest for their association with sustained virological response (SVR) when treating patients of genotype-1 hepatitis C virus (GT1-HCV) chronic infection with pegylated interferon and ribavirin (PegIFN/RBV). This study was to investigate the predictive power of *IL28B SNPs* for on-treatment responses and SVR in treatment-naïve patients with GT1-HCV chronic infection.

**Methodology/Principal Findings:**

We analyzed ten SNPs of *IL28B* in 191 treatment-naïve patients with GT1-HCV chronic infection who received PegIFN/RBV. In these patients, rapid virological response (RVR), early virological response (EVR) and SVR were achieved in 69.6%, 95.8% and 68.6% of the patients, respectively. Multivariate analysis (odds ratio; 95% confidence interval; P value) indicated age (0.96; 0.93–0.99; 0.012), low baseline viral load (4.65; 2.23–9.66; <0.001) and CC genotype of rs12979860 (7.74; 2.55–23.53; <0.001) but no other SNPs were independent predictors for SVR. In addition, none of the ten SNPs examined were associated with baseline viral load and stages of liver fibrosis. Regarding RVR, low baseline viral load (2.83; 1.40–5.73; 0.004) and CC genotype of rs12979860 (10.52; 3.45–32.04; <0.001) were two critical predictors. As for EVR, only CC genotype of rs12979860 (36.21; 6.68–196.38; <0.001) was the predictor. Similarly, for end of treatment response (ETR), CC genotype of rs12979860 (15.42; 4.62–51.18; <0.001) was the only predictor. For patients with RVR, only low baseline viral load (3.90; 1.57–9.68; 0.003) could predict the SVR. For patients without RVR, only rs12979860 (4.60; 1.13–18.65; 0.033) was the predictor for SVR.

**Conclusions/Significance:**

rs12979860 is the critical predictor for RVR, EVR, ETR and SVR in treatment-naïve patients of GT1-HCV chronic infection. Furthermore, this SNP is the only predictor for SVR in patients without RVR. These results have provided evidence that rs12979860 is the ideal *IL28B* SNP for genetic testing in treating patients of GT1-HCV chronic infection.

## Introduction

A combination therapy of pegylated interferon-alpha and ribavirin (PegIFN/RBV) is a well-accepted standard of care for patients with chronic hepatitis C (CHC) [1]. Current strategy for CHC treatment is to individualize the treatment duration guided by genotype and on-treatment viral response [1], [2]. With these recommended therapies, a sustained virological response (SVR) rate could reach 42–79% among HCV genotype 1 and 76–95% among HCV genotype 2/3 [2]. Interestingly, better response rates were found in Asian patients, with SVR rates around 61–79% for HCV genotype 1 patients [3], [4], [5], [6] and 80–95% for HCV genotype 2/3 patients [7], [8], [9]. As for the response-guided approach, rapid virological response (RVR) is regarded as an important predictor for SVR [5], [10], [11]. It is also a guide for shortening treatment duration from 48 weeks to 24 weeks for HCV genotype 1 with low viral load [4], [5], [12], [13] and from 24 weeks to 12–16 weeks for genotype 2/3 chronic infection [10], [14], [15]. In addition, early virological response (EVR) is an important parameter for the decision to terminate or continue treatment because patients without EVR could hardly achieve SVR[16]. On the other hand, undetectable virus at the end of treatment is coined as an end-of-treatment response (ETR). Though an ETR does not accurately predict whether an SVR will be achieved, it is necessary for SVR to occur[1].

Host genetic factors on the treatment efficacy for chronic hepatitis C were proposed a long time ago due to the ethnic differences in the treatment outcome. Recent genome-wide associated studies have explored this issue and demonstrated strong evidence that single nucleotide polymorphisms (SNPs) of *Interluekin*-*28B (IL28B)* were significantly correlated with SVR when patients were treated with PegIFN/RBV [17], [18], [19], [20], [21]. Notably, the frequency of advantageous C allele of rs12979860 of *IL28B* was reported highest in Asians and lowest in African-Americans. In addition, the prevalence rates of cc genotype of rs1297860 paralleled with the SVR in each population [17]. Furthermore, these genotypes of *IL28B* also correlated with the spontaneous clearance of hepatitis C virus [18] and with viral responses during treatment [21]. Because of its significant impact on the treatment outcome, a genetic testing for the genotype of SNP of *IL28B* before deciding on treatment strategies has been proposed [22], [23]. However, several other SNPs of *IL28B* were also found to be highly associated with SVR, like rs8099917 [20], rs12980275 [19], and others [17], [18], [19], [20]. Nevertheless, which SNP would be the most influential on SVR was still undetermined.

We investigated these issues and tried to increase our understanding of the predictive ability of 10 SNPs of *IL28B* for RVR, EVR and SVR in a cohort of patients with GT1-HCV chronic infection treated with PegIFN/RBV from a large medical center.

## Materials and Methods

### Patients

**Figure 1:**
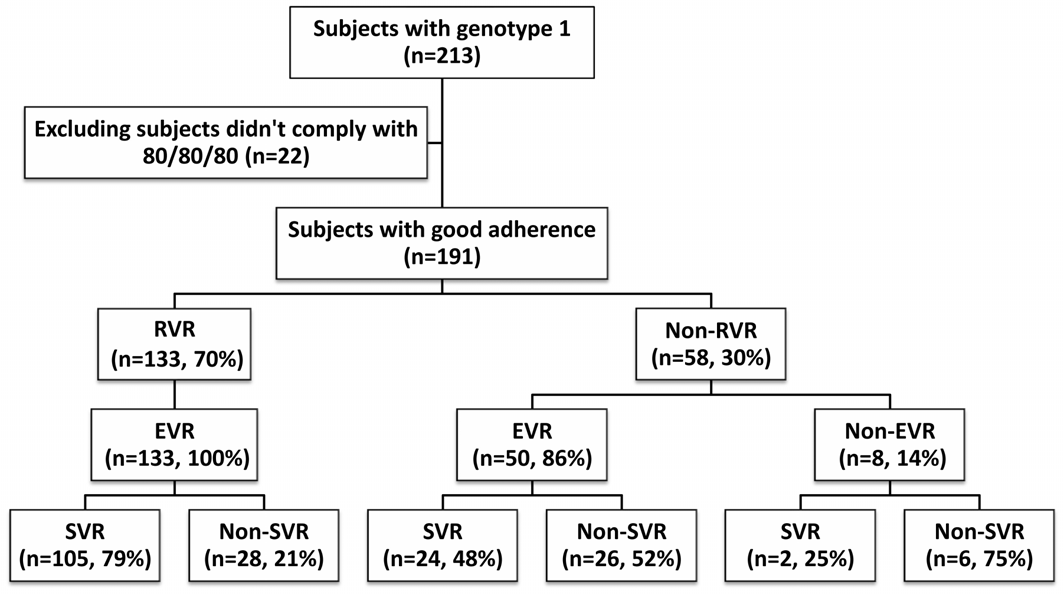
Flow diagram illustrating the selection of subiects for analysis and their treatment outcomes.

We analyzed a prospective cohort of 213 consecutive adult Taiwanese treatment-naïve patients with chronic hepatitis C virus genotype 1 who visited HCV team of Department of Gastroenterology and Hepatology, Linkou Medical Center, Chang Gung Memorial Hospital, and received 24 weeks of combination therapy with PegIFN/RBV between February 2002 and December 2008, and who agreed to provide blood samples for the human genome study. Patients with decompensated liver disease, hepatoma, co-infection with hepatitis B virus or with human immunodeficiency virus, with apparent autoimmune hepatitis and alcoholic liver disease were excluded from this cohort. All patients included in the study had received liver biopsies that were evaluated by one pathologist using the Metavir scoring system. HCV genotype was determined by a genotype specific probe based assay in the 5′ untranslated region (LiPA; Innogenetics, Ghent, Belgium). In these 213 patients, we excluded 22 patients who did not fit the 80/80/80 adherence rule (less than 80% of total PEG-IFN or total RBV doses or less than 80% of the total duration of therapy), with a final case number of 191 (Fig. 1).

Definitions of response to treatment were undetectable serum HCV-RNA levels 24 weeks after cessation of treatment as SVR, undetectable serum HCV-RNA levels at 4 week after starting treatment as RVR, at least two-log_10_ reduction of viral load at week 12 after starting treatment as EVR, and undetectable virus at the end of treatment as ETR.

The HCV-RNA levels in this study were measured using a commercial quantitative polymerase chain reaction (PCR) assay VERSANT HCV RNA 3.0. Assay (HCV 3.0 bDNA assay, Bayer Diagnostics, Berkeley, Calif., lower limit of detection: 5.2×10^2^IU/ml) or COBAS TaqMan HCV Test (TaqMan HCV; Roche Molecular Systems Inc., Branchburg, N.J., lower limit of detection: 15 IU/ml). If non-detection of HCV-RNA by VERSANT HCV RNA 3.0. Assay, it would be further tested by COBAS® AMPLICOR HCV Test, v2.0 (CA V2.0, Roche Diagnostic Systems, lower limit of detection: 50 IU/ml).

### Genomic DNA extraction and *IL28 B* Genotyping

Anti-coagulated peripheral blood was obtained from HCV patients. Genomic DNA was isolated from EDTA anti-coagulated peripheral blood using the Puregene DNA isolation kit (Gentra Systems, Minneapolis, MN) as previously described[24]. The oligonucleotide sequences flanking ten *IL28B* polymorphisms were designed as primers for Taqman allelic discrimination. The allele specific primers were labeled with a fluorescent dye (FAM and VIC) and used in the PCR reaction. Aliquots of the PCR product were genotyped with allele specific probe of SNPs using real time PCR (ABI). Ten SNPs of *IL28B* including rs12979860, rs11881222, rs4803219, rs8099917, rs12980275, rs8105790, rs7248668, rs10853728, rs8103142 and rs28416813 were chosen according to previous reports [17], [18], [19], [20].

### Ethics Statements

All patients in this study had provided written informed consent. This study protocol conformed to the ethical guidelines of the 1975 Declaration of Helsinki and was approved by the ethical committees of Chang Gung Memorial Hospital.

### Statistical analysis

Linkage disequilibrium (LD) between marker loci was assessed and haplotype blocks were constructed using Haploview 4.1. Chi-square tests and Fisher's exact probability tests were used as appropriate to compare the categorical variables of the groups. Continuous variables were expressed as means and standard deviations (SDs) and compared using Mann-Whitney U test or Student's *t*-test. Univariate and multivariate logistic regression analyses for predictors of sustained virological response were conducted using patients' demographic, clinical variables and *IL28B* SNPs. The clinical variables included gender, age, viral load of HCV-RNA, Metavir fibrosis stage, body mass index (BMI), Glycohemoglobin (HbA1c), albumin, aspartate transaminase, alanine transaminase, bilirubin, gamma-glutamyl transpeptidase and alkaline phosphatase.

The odds ratios (OR) and 95% confidence intervals (95% CI) were also calculated. Breslow-Day test and Cochran's-Mantel-Haenszel test were used to estimate the homogeneity of odds ratios (ORs) between stratified groups. All *P* values less than 0.05 by the two-tailed test were considered statistically significant. Variables that achieved a statistical significance less than 0.10 in the univariate analysis were entered into multivariate logistic regression analysis to identify the significant independent predictive factors. All statistical analyses were performed with statistical software SPSS for Windows (version 16, SPSS. Inc., Chicago, IL, USA).

## Results

### rs12979860 CC genotype is the most powerful predictor among SNPs of IL28B for RVR, EVR, ETR and SVR in chronic GT1 HCV infected patients treated with PegIFN/RBV

**Figure 2:**
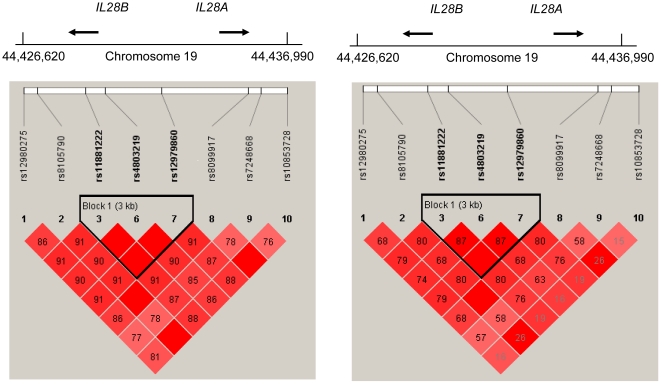
Pairwise linkage disequilibrium (LD) patterns for eight polymorphisms through *IL28B* regions (chromosome 19, nucleotide positions 44,426,620–44,436,990).

**Figure 3:**
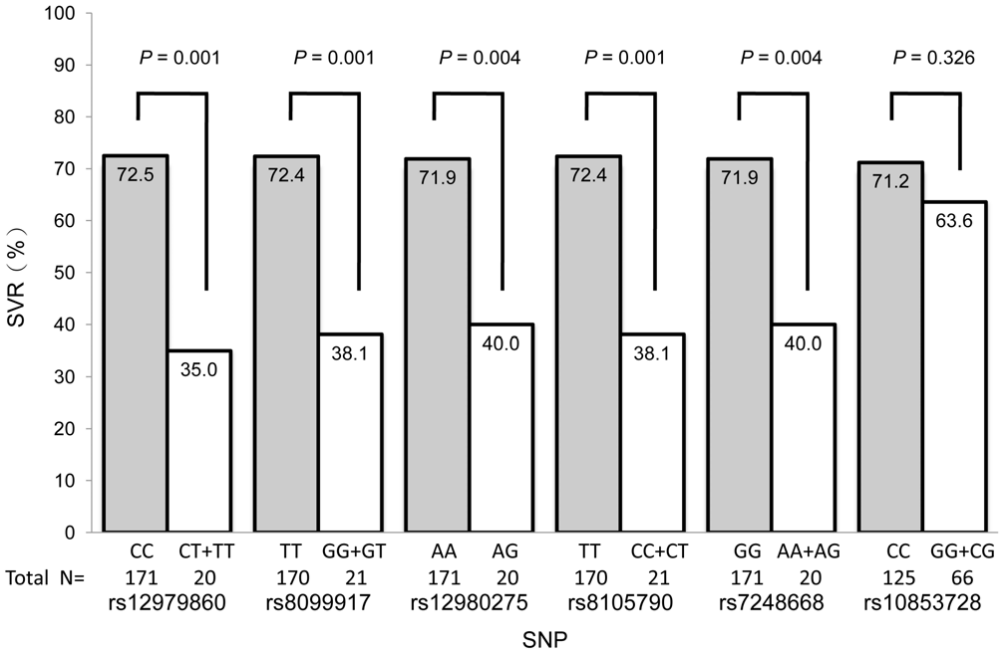
*IL28B* genetic association with sustained virological response in patients with GT1-HCV infection treated with PegIFN/RBV. Percentages of patients of different genotypes with SVR in eight different SNPs groups were shown. Numbers of patients were also shown below each genotype.

Demographic characteristics of 191 CHC genotype-1 (GT1) patients enrolled in this prospective cohort were shown in Table 1. Among these patients, 133 (69.63%) achieved RVR, 183 (95.81%) achieved EVR and 131 (68.59%) achieved SVR (Figure 1). From the result of statistic analysis, the factors favoring SVR were low baseline viral load (HCV-RNA <0.4×10^6^ IU/mL), less fibrosis stage (Metavir fibrosis score F0–F2), low body mass index (BMI), lower gamma-glutamyl transferase (GGT), RVR and EVR (Table 1). Ten SNPs of *IL28B* genetic variations were genotyped in each patient. Two loci, rs8103142 and rs28416813, were excluded from this analysis due to significant deviations from Hardy-Weinberg equilibrium in genotype and allele distributions. In addition, one haplotype block consisting of 3 SNPs, rs11881222, rs4803219 and rs12979860, was identified due to strong evidence of linkage disequilibrium (Figure 2) and rs12979860 was chosen to represent this haplotype. In all the six SNPs under analysis, five SNPs were significantly associated with SVR except rs10853728 (Figure 3).

**Table 1:** Baseline characteristics of genotype I chronic hepatitis C patients treated with PegIFN/RBV and sustained virological response.

Variable	Total(N = 191)	Non-SVR(N = 60)	SVR(N = 131)	P value
Sex, n (%)				0.131
male	123 (64.4)	26 (43.3)	42 (32.1)	
female	68 (35.6)	34 (56.7)	89 (67.9)	
Baseline Viral Load (mean ± SD)				<0.001
<0.4×10^6^ IU/ml	91 (47.6)	16 (26.7)	75 (57.3)	
≥0.4×10^6^ IU/ml	100 (52.4)	44 (73.3)	56 (42.7)	
Fibrosis Stage, n (%)				0.021
F0-2	103 (53.9)	25 (41.7)	78 (59.5)	
F3-4	88 (46.1)	35 (58.3)	53 (40.5)	
Age, *y* (mean ± SD)	51.4±11.1	53.6±10.3	50.3±11.4	0.063
BMI, *Kg/m^2^* (mean ± SD)	24.5±3.3	25.2±3.1	24.2±3.4	0.049
HbA1c, *%* (mean ± SD)	5.6±1.0	5.7±1.1	5.6±1.0	0.443
ALB, *g/dL* (mean ± SD)	4.5±0.4	4.4±0.4	4.5±0.3	0.507
AST, *IU/L* (mean ± SD)	92.0±51.6	89.6±2.0	93.1±51.5	0.532
ALT, *IU/L* (mean ± SD)	149.1±107.2	129.4±1.2	158.2±116.3	0.068
GGT, *IU/L* (mean ± SD)	42.9±48.5	51.9±60.0	38.8±41.9	0.034
ALP, *IU/L* (mean ± SD)	79.5±25.2	81.9±27.6	78.3±24.0	0.443
Bilirubin, *mg/dL* (mean ± SD)	0.9±0.3	1.0±0.3	0.9±0.3	0.213
RVR, n (%)	133 (69.6)	28 (46.7)	105 (80.2)	<0.001
EVR, n (%)	183 (95.8)	54 (90.0)	129 (98.5)	0.007

BMI: body mass index. ALB: albumin; AST: aspartate transaminase; ALT: alanine transaminase; GGT: gamma-glutamyl transpeptidase; ALP: alkaline phosphatase; SVR: sustained viralogical response; RVR: rapid virological response; EVR: early virological response.

We then examined the predictive ability of each SNP and other clinical baseline parameters for SVR by logistic regression (Table 2). Interestingly, only the rs12972860 CC genotype, but not other SNPs, together with younger age and low baseline viral load (<0.4×10^6^ IU/ml) became the significant predictors for SVR by the multivariate analysis (Table 2). However, TT genotype of rs8099917 was also known as another important predictor for SVR [19], [20], [25], [26], [27], [28], [29]. We reasoned that the impact of rs12979860 was too strong to mask the effect of genotype of rs8099917 on SVR. Therefore, we omitted the data of rs12979860 and re-analyzed. As shown in Table 3, without the factor of rs12979860, the TT genotype of rs8099917, together with younger age and low baseline viral load emerged the significant predictors for SVR by the multivariate analysis. As for the RVR, it is also the same SNP, rs12972860, and baseline viral load could predict RVR by multivariate analyses (Table 4). For the EVR and ETR, only rs12979860 was the predictor but not the baseline viral load (Table 5 and 6).

**Table 2:** Factors predicted SVR to Peg-IFN/RBV treatment in genotype I chronic hepatitis C patients by univariate and multivariate Logistic regression analysis.

Baseline Predictors		Odds Ratio	95% CI	p value
**Univariable**	Age (years old)	0.97	0.95–1.00	0.064
	Baseline Viral Load	3.68	1.89–7.19	<0.001
	Fibrosis stage (Metavir)	2.06	1.11–3.83	0.002
	BMI (Kg/m^2^)	0.92	0.83–1.00	0.060
	ALT (IU/L)	1.00	0.99–1.01	0.090
	GGT (IU/L)	1.00	0.99–1.00	0.109
	rs12979860	4.90	1.84–13.03	0.001
	rs8099917	4.25	1.66–10.92	0.003
	rs12980275	3.84	1.48–9.99	0.006
	rs8105790	4.25	1.66–10.92	0.003
	rs7248668	3.84	1.48–9.99	0.006
	rs10853728	1.41	0.75–2.66	0.285
**Multivariable**	Age (years old)	0.96	0.93–0.99	0.012
	Baseline Viral Load			
	≥0.4×10^6^ IU/ml	1		
	<0.4×10^6^ IU/ml	4.65	2.23–9.66	<0.001
	rs12979860			
	CT/TT genotype	1		
	CC genotype	7.74	2.55–23.53	<0.001

UV: univariate logistic regression analysis. MV: multivariate logistic regression analysis.

OR: odds ratio; CI: confidence interval. Fibrosis stage: Metavir scoring system.

**Table 3:** Factors predicted SVR to Peg-IFN/RBV treatment in genotype I chronic hepatitis C patients by univariate and multivariate Logistic regression analysis excluding rs12979860.

Baseline Predictors		Odds Ratio	95% CI	p value
**Multivariable**	Age (years old)	0.96	0.93–0.99	0.014
	Baseline Viral Load			
	≥0.4×10^6^ IU/ml	1		
	<0.4×10^6^ IU/ml	4.42	2.15–9.08	<0.001
	rs8099917			
	GG/GT genotype	1		
	TT genotype	6.11	2.12–17.64	0.001

**Table 4:** Factors predicted RVR to Peg-IFN/RBV treatment in genotype I chronic hepatitis C patients by univariate and multivariate Logistic regression analysis.

Baseline Predictors		Odds Ratio	95% CI	p value
**Univariable**	Baseline Viral Load	2.42	1.27–4.62	0.007
	HbA1c (%)	0.74	0.55–0.99	0.046
	rs12979860	8.93	3.07–26.02	<0.001
	rs8099917	4.51	1.76–11.61	0.002
	rs12980275	6.74	2.44–18.60	<0.001
	rs8105790	4.51	1.76–11.61	0.002
	rs7248668	6.74	2.44–18.60	<0.001
	rs10853728	1.53	0.81–2.89	0.192
**Multivariable**	Baseline Viral Load			
	≥0.4×10^6^ IU/ml	1		
	<0.4×10^6^ IU/ml	2.83	1.40–5.73	0.004
	rs12979860			
	CT/TT genotype	1		
	CC genotype	10.52	3.45–32.04	<0.001

**Table 5:** Factors predicted EVR to Peg-IFN/RBV treatment in genotype I chronic hepatitis C patients by univariate and multivariate Logistic regression analysis.

Baseline Predictors		Odds Ratio	95% CI	p value
**Univariable**	HbA1c (%)	0.63	0.41–0.97	0.034
	GGT (IU/L)	0.98	0.98–0.99	0.002
	rs12979860	36.21	6.68–196.38	<0.001
	rs8099917	9.77	2.24–42.60	0.002
	rs12980275	18.67	4.06–85.84	<0.001
	rs8105790	9.77	2.24–42.60	0.002
	rs7248668	36.21	6.68–196.38	<0.001
	rs10853728	1.95	0.47–8.07	0.356
**Multivariable**	rs12979860			
	CT/TT genotype	1		
	CC genotype	36.21	6.68–196.38	<0.001

**Table 6:** Factors predicted ETR to Peg-IFN/RBV treatment in genotype I chronic hepatitis C patients by univariate and multivariate Logistic regression analysis.

Baseline Predictors		Odds Ratio	95% CI	p value
**Univariable**	Sex	2.53	0.90–7.13	0.079
	BMI (Kg/m^2^)	0.88	0.77–1.02	0.081
	GGT (IU/L)	0.99	0.98–0.99	0.018
	rs12979860	13.58	4.34–42.56	<0.001
	rs8099917	8.94	2.89–27.65	<0.001
	rs12980275	6.90	2.19–21.79	0.001
	rs8105790	8.94	2.89–27.65	<0.001
	rs7248668	13.58	4.34–42.56	<0.001
	rs10853728	2.66	0.94–7.51	0.064
**Multivariable**	Sex			
	female	1		
	male	3.12	0.98–9.95	0.055
	rs12979860			
	CT/TT genotype	1		
	CC genotype	15.42	4.64–51.18	<0.001

### rs12979860 CC genotype had a significant impact on the SVR irrespective to baseline viral load

As shown in Table 2, baseline viral load and *IL28B* genotypes are the two important predictors of the SVR when treated with PegIFN/RBV. Because *IL28B* is one of the cytokines that play an important role in anti-viral immunity, we then analyzed the relationship between these SNPs and the baseline viral load and stages of liver fibrosis. As show in Table 7, none of these SNPs correlated with the baseline viral load and fibrosis stage. We also studied the influence of the *IL28B* genotype on the SVR in patients with either high baseline viral load or low baseline viral load. Because rs12979860 is the single statistically significant *IL28B* predictor of SVR as shown previously, we focused on this locus in the groups of either high or low baseline viral load. As shown in Table 8, the genotype of rs12979860 was significantly associated with the SVR in both groups of high baseline viral load or low baseline viral load. Furthermore, the odds ratios between these two groups had no difference (Odds ratio, low viral load *vs.* high viral load: 6.37 *vs.*5.99, p = 0.956, by Cochran's and Mantel-Haenszel statistics). Therefore, in addition to the findings that this SNP had no relationship with baseline viral load, the impact of the SNP on SVR remained similar either in the group of high baseline viral load or low baseline viral load.

**Table 7:** Influences of SNPs of *IL28B* on the baseline viral load and liver fibrosis.

Genotypes of SNPs		Baseline Viral load (IU/ml)			Fibrosis stage (Metavir)		
		<0.4×10^6^	≥0.4×10^6^	*P* value	F0-F2	F3-F4	*P* value
rs12979860	CC	81	90	0.824	90	81	0.348
	CT+TT	10	10		13	7	
rs8099917	GG+GT	81	89	0.998	88	82	0.088
	TT	10	11		15	6	
rs12980275	AA	82	89	0.818	90	81	0.294
	AG	9	11		13	7	
rs8105790	TT	81	89	1.000	88	82	0.088
	CC+CT	10	11		15	6	
rs7248668	GG	80	91	0.637	91	80	0.565
	AA+AG	11	9		12	8	
rs10853728	CC	59	66	0.880	64	61	0.298
	CG+GG	32	34		39	27	

**Table 8:** Influence of genotypes of rs12979860 on SVR in high and low baseline viral load.

Baseline Viral load	rs12979860	Non-SVRN (%)	SVRN (%)	OR(95% CI)	Homogeneity of OR^a^
Low viral load(<0.4×10^6^ IU/ml)	CC	11 (13.6)	70 (86.4)	6.37 (1.6–25.6)*p* = 0.013	*p* = 0.956
	CT/TT	5 (50.0)	5 (50.0)		
High viral load(≥0.4×10^6^ IU/ml)	CC	36 (40.0)	54 (60.0)	5.99 (1.2–30.3)*p* = 0.020	
	CT/TT	8 (80.0)	2 (20.0)		

a Cochrans and Mantel-Haenszel statistics.

### rs12979860 CC genotype could not predict SVR in patients with RVR but could in patients without RVR

RVR is an important predictor for SVR and a useful guide in treating patients with chronic hepatitis C [1], [2]. In the present study, for patients with RVR, SVR was achieved in 79.0% of the instances, significantly higher than patients without RVR (SVR: 44.83%, *P* <0.001). We evaluated the influence of these SNPs on the SVR in these two groups of patients. In patients with RVR, only low baseline viral load was a significant predictor for SVR but not any SNPs of *IL28B* or other clinical parameters (Table 9). On the contrary, in patients without RVR, only CC genotype of rs12979860 could predict the SVR but not other clinical parameters including baseline viral load (Table 10).

**Table 9:** Factors predicted SVR to Peg-IFN/RBV treatment in patients with RVR by univariate and multivariate Logistic regression analysis.

Baseline Predictors		Odds Ratio	95% CI	p value
**Univariable**	Age (years old)	0.97	0.93–1.01	0.157
	Baseline Viral Load	3.90	1.57–9.68	0.003
	Fibrosis Stage (Metavir)	2.70	1.14–6.42	0.025
	BMI (Kg/m^2^)	0.96	0.85–1.08	0.459
	ALT (IU/L)	1.01	1.00–1.01	0.058
	GGT (IU/L)	1.00	0.98–1.02	0.938
	rs12979860	0.94	0.10–8.72	0.953
	rs8099917	2.40	0.54–10.72	0.252
	rs12980275	0.74	0.08–6.61	0.788
	rs8105790	2.40	0.54–10.72	0.252
	rs7248668	1.94	0.34–11.19	0.457
	rs10853728	0.84	0.33–2.09	0.700
**Multivariable**	HCV RNA levels			
	≥0.4×10^6^ IU/ml	1		
	<0.4×10^6^ IU/ml	3.90	1.57–9.68	0.003

**Table 10:** Factors predicted SVR to Peg-IFN/RBV treatment in patients without RVR by univariate and multivariate logistic regression analysis.

Baseline Predictors		Odds Ratio	95% CI	p value
**Univariable**	Age (years old)	0.97	0.92–1.02	0.170
	Baseline Viral Load	2.20	0.72–6.72	0.166
	Fibrosis Stage (Metavir)	1.55	0.55–4.39	0.413
	BMI (Kg/m^2^)	0.84	0.69–1.02	0.080
	ALT (IU/L)	1.00	0.99–1.00	0.894
	GGT (IU/L)	0.99	0.99–1.00	0.519
	rs12979860	4.60	1.13–18.65	0.033
	rs8099917	3.49	0.85–14.37	0.084
	rs12980275	4.02	0.98–16.40	0.053
	rs8105790	3.49	0.85–14.37	0.084
	rs7248668	2.50	0.68–9.19	0.168
	rs10853728	2.25	0.76–6.65	0.142
**Multivariable**	rs12979860			
	CT/TT genotype	1		
	CC genotype	4.60	1.13–18.65	0.033

Taken together, we identified rs12979860, one of the 10 SNPs of *IL28B*, to be the most powerful predictor for RVR, EVR, ETR and SVR in GT1 HCV infected patients treated with PegIFN/RBV. In addition, rs12979860 genotype could predict the SVR in patients without RVR but could not in patients with RVR.

## Discussion

Four seminal papers had recently revealed a significant impact of *IL28B* polymorphisms on the treatment outcome of patients with chronic hepatitis C [17], [18], [19], [20]. Subsequently, several investigators also published similar observations in different populations different loci of SNPs of *IL28B* [21], [25], [26], [27], [28], [29], [30], [31], [32], [33], [34], [35]. Herein, we extended these observations and demonstrated that rs12972860 is the most powerful SNP predictor for RVR, EVR, ETR and SVR in patients of CHC genotype 1 treated with PegIFN/RBV. Furthermore, we also demonstrated that this SNP could only predict the SVR in patients without RVR but could not predict the SVR in patients with RVR.

Based on previous reports on the SNPs of *IL28B* [17], [18], [19], [20], we had genotyped ten different SNPs of *IL28B* in our patient cohort. These ten SNPs were most strongly associated with SVR and were also found at the *IFN-λ* gene cluster [17], [18], [19], [20]. We excluded two loci, rs8103142 and rs28416813, from analysis due to significant deviations from Hardy-Weinberg equilibrium in genotype and allele distributions. One of these two loci, rs8103142, was a nonsynonymous SNP, within the *IL28B* gene that encodes a lysine to arginine substitution at position 70 (K70R). Another locus, rs28416813, was a G to C substitution at 37 base pairs upstream of the translation initiation site[23]. In addition, we also found one haplotype block and we chose rs12979860 to represent this haplotype because this locus was frequently reported in previous literatures [17], [18], [29], [30], [31], [32], [33], [34], [35]. In total, 5 SNPs were significantly correlated with the SVR except one SNP, rs10853728, which was not. This SNP, rs10853728, had been reported to be associated with SVR in Japanese population with GT1 HCV infection [19] but was not associated with RVR and SVR in Taiwanese patients with GT2/3 HCV infection [36].

However, we found the genotype of rs12979860, but not other SNPs, together with age and viral load became the predictors of SVR. rs12979860 is the frequently mentioned SNP of IL28B related to SVR [17], [18], [29], [30], [31], [32], [33], [34], [35], and represents a haplotype block. rs8099917 was another frequently reported SNP for SVR in GT1 HCV infected patients [19], [20], [25], [26], [27], [28]. rs12979860 and rs8099917 reside 3 and 8 kilobases, respectively, upstream of the IL28B gene encoding IFN-λ-3 [23]. In our analysis, rs8099917 became the predictor for SVR only after omitting the data of rs12979860. Therefore, though several SNPs of IL28B had been claimed to be important for predicting SVR, it is clear the rs12979860 is the key predictor among these SNPs for SVR in GT1 HCV infected patients.

Concerning the relationship between baseline viral load and SNPs of *IL28B*, the results from previous reports were not consistent. Some reports claimed the baseline viral load was correlated with the genotype of the SNP but others did not [17], [28], [32], [34], [37]. Our data showed no relationship between baseline viral load and the SNPs. In addition, we also demonstrated that in both groups of high viral load and low viral load, SNP of *IL28B* could predict the SVR with similar predictive ability. Therefore, in our cohort, SNP of *IL28B* was only related to the treatment outcome but was not related to the viral load.

A customized therapy for chronic hepatitis C based on genotype and treatment viral response is the current treatment strategy [1], [2]. RVR is a useful predictor of SVR in treatment of chronic hepatitis C with either conventional IFN or PegIFN plus RBV therapies [5], [10], [11], [38]. As for the predictor of RVR, previous reports have shown baseline viral load was the single predictor [38], or together with younger age, lower body weight and absence of advanced fibrosis as predictors [5], [13], [39]. In the present study, we found baseline viral load was the only clinical parameter as the predictor for RVR. As for the SNPs of IL28B, it is again the rs12979860, but not other SNPs, that was the predictor for RVR. On the other hand, EVR was also an important negative response guider for treatment[16]. In previous reports, only the baseline viral load could predict the EVR [40]. In the present analysis, only rs12979860 but not other SNPs nor other clinical parameters, including baseline viral load, was the predictor for EVR. These results again emphasized the significant impact of this SNP to the treatment responses. Taken together, only the genotype of rs12979860 is the predictor for RVR, EVR, ETR and SVR. These results are consistent with the idea that SNPs of *IL28B* is related to the immune responses after interferon-plus RBV treatment [41].

Another interesting observation in our study was the different impact of the CC genotype of rs12979860 on the SVR in patients with or without RVR. In patients with RVR, only baseline viral load but not the genotype of rs12979860 could predict the SVR. This is an interesting observation. The possible explanation is the CC genotype of rs12979860 led to the RVR with odds ratio of 10.52. As a result, the majority of patients with RVR were CC genotypes. Therefore, the CC genotype lost their predictive ability for SVR in this patient group with high prevalence of CC genotype. On the contrary, in patients without RVR, only the genotype of rs12979860 but not the baseline viral load could predict the SVR. This group of patients without RVR was with lower prevalence of CC genotype. Therefore, the CC genotype could continue its influence and therefore predict the SVR in this group of patients. This observation was quite similar to the recent report about genotype 2/3 HCV infected patients who received treatment that the genotype of rs12979860 is a single predictor for SVR in patients without RVR [35].

Since the discovery of the strong association between SNPs of IL28B and treatment outcome of chronic hepatitis C, a consideration of personalized approach for treatment of chronic hepatitis C based on these SNPs had been proposed [22]. As suggested by Dr. Afdhal et al, although IL28B genotyping is highly predictive of SVR in HCV GT1 infected patients, its predictive power at the individual patient level is far from absolute. Therefore, IL28B genotyping should not be the sole factor in deciding on a treatment strategy [45]. In addition, shortening of treatment duration based on these SNPs together with other clinical parameter is possible but there are insufficient data to make recommendations [45]. However, the predictive ability of IL28B genotypes is strong enough. Therefore, it is reasonable to recommend a pre-treatment genotyping for SVR prediction. Based on our results described previously, we suggested genotyping for rs12979860 rather than other SNPs of *IL28B* would be a suitable genetic testing before starting treatment for patients with GT1-HCV chronic infection.

On the other hand, though a strong association between IL28B polymorphism and SVR had been repeated reported, the underlying mechanisms are still unclear. Recent in-vitro report had shown that IL28B could inhibit HCV replication in a dose- and time- dependent manner and through the JAK-STAT pathway [42]. Consequently, it had been found that IL28B genotype is associated with differential expression of intrahepatic interferon-stimulated genes in patients with chronic hepatitis C [43]. Furthermore, serum IL-28A/B levels were significantly higher in patients with chronic hepatitis C with good allele of IL28B genotype [44]. All these evidences indicate both direct anti-viral effect and immune-mediated effect of IL28B could be affected by these polymorphisms. However, detailed mechanistic understanding needs further investigation.

The limitation of this study was the retrospective nature of the analysis. However, it is common to all the papers published on this issue. The other limitation was that we had excluded the patients who didn't adhere to the 80/80/80 principle in order to investigate the real effects of SNPs on the treatment response. However, this exclusion would possibly ignore the possibilities that some patients without 80/80/80 adherence were due to the effect of these SNPs though till now there was no such report. Prospective controlled studies would be necessary to evaluate the influence of different SNPs of IL28B gene, especially rs12979869 and rs8077717, on treatment outcome of patients with chronic hepatitis C.

In conclusion, our data demonstrated that the genotype of rs12979860 is the most powerful predictor for RVR, EVR, ETR and SVR among ten SNPs of *IL28B* in patients with GT1 HCV infection treated with PegIFN/RBV. Furthermore, we also demonstrated different impact of baseline viral load and genotype of rs12979860 on SVR in patients with or without RVR. Based on these results, we suggested that the genotyping of rs12979860 is the suitable target for genetic testing before treatment for patients with GT1-HCV chronic infection.
